# Factors associated with high HIV-related stigma among commuter populations in Johannesburg, South Africa

**DOI:** 10.1080/17290376.2021.1989022

**Published:** 2021-10-26

**Authors:** Peter S. Nyasulu, Ndumiso Tshuma, Lovemore N. Sigwadhi, Juliet Nyasulu, Modupe Ogunrombi, Lucy Chimoyi

**Affiliations:** a Division of Epidemiology & Biostatistics, Faculty of Medicine & Health Sciences, Stellenbosch University, Cape Town, South Africa; b Best Health Solutions, Orange Grove, Johannesburg, South Africa; c Division of Community Paediatrics, School of Clinical Medicine, Faculty of Health Sciences, University of the Witwatersrand, Johannesburg, South Africa; d Department of Clinical Pharmacology, School of Medicine, Sefako Makgatho Health Science University, Pretoria, South Africa; e The Aurum Institute, Johannesburg, South Africa; f School of Public Health, Faculty of Health Sciences, University of the Witwatersrand, Johannesburg, South Africa

**Keywords:** HIV, stigma, HCT, commuter populations, Johannesburg, South Africa

## Abstract

Stigma remains an important barrier to seeking and staying in care among individuals infected with Human Immunodeficiency Virus (HIV). Despite continued widespread information, education and communication campaigns to raise awareness about the infection. The aim of the study was to identify factors related to HIV stigma among a commuter population in the inner-city Johannesburg. A self-administered closed-ended questionnaire was loaded onto personal tablet computers during a community outreach campaign. The outcome was measured by asking the respondents to rate their perceptions of stigma as ‘high or low’. About 1146 participants were enrolled in the study of which 585 (51.0%) reported high stigma levels. Overall, being married/cohabiting (Adjusted Prevalence Ratio (APR): 1.14 95%CI: 1.02–1.28), divorced (APR: 1.38 95%CI: 1.07–1.78), were associated with high levels of stigma; while being aware of HCT services (APR: 0.85 95%CI: 0.75–0.97) and employment status (APR: 0.78 95%CI: 0.71–0.87) were less likely associated with a high level of stigma. High HIV stigma still exists among those affected in our communities. Enhancement of health promotion intervention and reinforcing the benefits of knowing HIV status is essential to mitigate factors shown to influence stigma in the commuter population. Such an approach would help overcome stigma, an obstacle for expanding access to HIV testing and counselling.

## Introduction

The United Nations Programme on HIV/AIDS (UNAIDS) developed a global strategy to end Human Immunodeficiency Virus/Acquired Immunodeficiency Syndrome (HIV/AIDS) that sets out parameters for all countries to increase HIV testing and access to treatment [UNAIDS, 2017]. These targets elucidate that by the year 2020, 90% of people with HIV infection are aware of their status, 90% of those aware are accessing antiretroviral treatment and among those on antiretroviral treatments, 90% are virologically suppressed [UNAIDS, 2017]. However, to attain these 90–90–90 goals, it is crucial to understand obstacles to HIV testing and treatment services. Research on obstacles to HIV testing has repeatedly highlighted high stigma and discrimination against people living with HIV infection (PLHIV) (Ekstrand et al., 2018; Hargreaves et al., 2018). Stigma, whether external (the actual experience of discrimination by unfair treatment) or internal (felt or imagined shame and expectation of discrimination) prevents individuals from disclosing their status and stopping them from seeking healthcare services (Mbonu et al., 2009)

Stigma remains the single most important barrier to seeking HIV care and treatment in many HIV infected individuals and as a result, the HIV/AIDS epidemic continues to devastate societies around the world (Akande, 2009; Alemu et al., 2013; dos Santos et al., 2014; Jain et al., 2013; Muloongo et al., 2014; Pitpitan et al., 2012; Turan et al., 2011; Zou et al., 2013). Studies in South Africa revealed that HIV stigma (Gilbert & Walker, 2010) is a significant barrier to HIV testing (Mwamburi et al., 2005) as it is known to interfere with HIV counselling, prevention, diagnosis, and treatment efforts (Jürgensen et al., 2013; Mall et al., 2013; Meyerson et al., 2014; Simbayi et al., 2007; Stangl et al., 2013; Visser et al., 2009). The fact that stigma is a social construct; individuals infected with HIV are more likely to experience a shift in attitude from their partners, family and friends (Alonzo & Reynolds, 1995). Fear of stigmatisation, discrimination and breach of confidentiality result in low uptake of HIV testing services (HTS) (Zou et al., 2013; Krause et al., 2013; Shroufi et al., 2013; Zhang et al., 2014). Furthermore, stigma prevents people from disclosing their status from public view resulting in reduced need for behavioural change (Alemu et al., 2013). Other studies have also showed that stigma is of utmost concern because it is both the cause and effect of secrecy and denial, which perpetuate HIV transmission (Mall et al., 2013; Sengupta et al., 2011).

Improving uptake of HIV testing requires a continual need to address HIV-related stigma (Musheke et al., 2013). Internationally, there has been an interest in HIV/AIDS-related stigma and discrimination triggered at least in part by a growing recognition that negative social responses to the epidemic remain pervasive even in seriously affected communities (Parker & Aggleton, 2003). As such, targeted and innovative approaches, which factor in the reduction of stigma and discrimination need to be designed to increase HIV testing rates.

This study focused on the commuter population who are a group of people that travel long distances to and from work or in search of work on a regular basis (Muloongo et al., 2014). This group of people who transit through a central transport terminus on a daily basis may not regularly seek primary health care services including HIV testing services, as these services are not readily available at transport terminuses and their workplaces. As such, the transport terminus provides an ideal site through which the government’s efforts of expanding access to HIV testing services were complemented. The success of such programs would require understanding the underlying reasons for HIV/AIDS-related stigma. This study therefore aimed to investigate factors associated with HIV/AIDS-related stigma among the commuter population in order to enhance uptake of HTS services among this group.

## Materials and methods

### Study design

A cross sectional survey was employed that adopted a venue-based intercept approach which involved recruiting participants at a Taxi transit point in Johannesburg’s inner-city central business district (CBD). Participants were selected using a simple random convenient sampling method (Chimoyi et al., 2015).

### Study setting

The study was conducted at the Noord Street taxi rank in Johannesburg, South Africa, which is in the heart of the CBD (COJ, 2013). Due to high migration rates and small business operations, the CBD has become a very vibrant and densely populated city with a diverse and cosmopolitan population. The increase in population size has been due to an influx of people to Johannesburg in search of jobs in the mining sector and small business operations (Ayala et al., 2010). The Noord Street taxi rank is Johannesburg’s primary transit point from different townships, the city periphery and for people dwelling in the CBD itself. The CBD is densely populated with no tangible housing options other than the illegal, informal renting spaces, which often take place out of dilapidated old buildings (Ayala et al., 2010).

### Data collection

Data were collected using self-administered closed-ended questionnaire loaded onto personal tablet computers during a community outreach campaign. This was organised by the Department of Health and it targeted individuals in and around taxi ranks in Johannesburg CBD. The survey staff approached and recruited individuals in and around the taxi rank. Those who agreed to participate were asked to provide the following information. Socio-demographic characteristics: gender categorised as (male, female), age (≤25; >25 years), marital status (single, cohabitating/married, divorced), employment status (employed, unemployed), educational level (primary, secondary, tertiary), sexual partnerships (none, one, >one), affected by HIV (no, yes), tested for HIV (no, yes), last HIV test (never tested, less than a year ago, more than a year ago), preferred testing place (clinic/hospital, home/mobile outreach).

### Measures

All measures were based on self-report. The outcome measure was determined by asking the respondents to rate their level of stigma as ‘High’ or ‘Low’. Details of additional measures used in this study have been reported elsewhere (Muloongo et al., 2014; Tshuma et al., 2015).

### Statistical analysis

Data were analysed using STATA version 15.1 (StataCorp, College Station, TX, USA). The analysis examined the relationship between the outcome variable of interest (levels of stigma) and the independent variables, which were ‘socio-demographic, awareness, knowledge and perceptions of societal testing’. Complete case analysis was done and the analysis followed a three-step process: descriptive statistics, univariate analysis, and multivariate analysis. Descriptive analysis described the characteristics of the study participants and the comparisons of HIV-related knowledge and perceptions, stigma and health factors affecting the commuter population according to stigma level. The Chi-square test of independence was used to examine the differences between these groups. The Chi-square test is a two-tailed test, and the value of Chi-square is always positive. The Chi-square test assesses if there is a relationship or not between two independent variables. It is valid when no expected number is less than 1 and not more than twenty percent of expected values are less than five. Due to the high prevalence of stigma (51.1%) and the type of study, the odds ratio is not the best effect measure to report and prevalence ratio (PR) is more interpretable and easier to communicate to non-specialists than odds ratio. The odds ratio overestimates the PR when the outcome of interest is common (>10%) and provides wide confidence intervals (CIs) (Barros & Hirakata, 2003; Martinez et al., 2017; Richardson et al., 2015). Univariate log-binomial regression analyses were done to identify factors associated with high HIV stigma levels. PRs and corresponding 95% CIs were used to determine statistically significant factors associated with high stigma levels. The log-binomial faced convergence challenges when the number of continuous independent variables was increased and robust Poisson regression was used as an alternative approach (Greenland, 2004; McNutt, 2003; Skov et al., 1998; Zou, 2004). Multivariate robust Poisson regression analyses were done using individual-level predictors that had *p* < 0.01 in the univariate analyses. Factors with *p* < 0.05 in multivariate analysis were considered to be independently associated with high HIV stigma levels. Deviance residuals and Hosmer-Lemeshow test were used to assess the model fit. Receiver operating curve (ROC) and area under the curve (AUC) were used to assess the model performance and the ability of the model to discriminate high and low stigma. Variance inflation factor and tolerance were used to assess multicollinearity between independent variables.

## Results

Basic descriptive statistics are given in Table 1. In a sample of 1146 respondents from the study area, 585 (51.0%) reported a high level of stigma. The proportion of respondents who preferred testing at home or through mobile outreach centres was higher than those who preferred testing in a health facility such as a clinic or hospital. Table 1 shows that 74.2% of the individuals who reported low HIV-related stigma were those whose perception of risk of HIV infection was low. A higher percentage of respondents cited ignorance 315 (59.3%) as a reason for high HIV-related stigma and discrimination.

**Table 1.** Characteristics of the study participants by stigma level.

Factor	Total N	High stigma n/N (%)	Low stigma n/N (%)	*p*-value*
Gender				
Female	579	309(52.8)	270(48.1)	0.112
Male	567	276(47.2)	291(51.9)	
Age group				
<25 years	366	192(32.8)	174(31.0)	0.512
≥25 years	780	393(37.2)	387(69.0)	
Marital status				
Single	801	402(68.7)	399(71.1)	0.056
Cohabiting/married	321	165(28.2)	156(27.8)	
Divorced	24	18(3.1)	6(1.1)	
Employment status				
Unemployed	474	273(46.7)	201(35.8)	<0.001
Employed	672	312(53.3)	360(64.2)	
Education level				
Primary	324	144(24.6)	180(32.1)	0.008
Secondary	498	258(44.1)	240(42.8)	
Tertiary	324	183(31.3)	141(25.1)	
Sexual partnerships				
None	108	51(8.7)	57(10.1)	0.629
One	681	354(60.5)	327(58.3)	
More than one	357	180(30.8)	177(31.6)	
Tested for HIV				
No	246	123(21.0)	123(21.9)	0.711
Yes	900	462(79.0)	438(78.1)	
Last HIV test				
Never tested	220	103(17.6)	117(20.9)	0.097
Less than a year ago	639	321(54.9)	318(56.7)	
More than a year ago	287	161(27.5)	126(22.4)	
Preferred testing place				
Clinic/hospital	414	198(33.9)	216(38.5)	0.101
Home/mobile outreach	732	387(66.2)	345(61.5)	
HCT awareness				0.035
No	906	477 (81.54)	429 (76.47)	
Yes	240	108 (18.46)	132 (23.53)	
Perceived benefit of HIV testing				<0.001
No	738	456 (77.95)	282 (50.27)	
Yes	408	129 (22.05)	279 (49.73)	
Health worker effect				0.014
None	135	57 (9.74)	78 (13.90)	
Doctors	243	111 (18.97)	132 (23.53)	
Counsellors	657	354 (60.51)	303 (54.01)	
Nurses	111	63 (10.77)	48 (8.56)	

*Bivariate associations determined by chi-square tests at 5% significance level.

Table 2 displays the results of univariate log-binomial regression with HIV-related stigma level as the outcome variable. Individuals who were divorced (*p* = 0.001), those with a secondary school education (*p* = 0.043), tertiary education (*p* = 0.002) were more likely to report high HIV-related stigma levels. Similarly, compared to individuals who had reported low HIV-related stigma levels, those who cited doctors and nurses as health workers responsible for reducing their willingness to test for HIV were significantly less likely to report high HIV-related stigma levels. Participants who were in employment were less likely to report high levels of HIV-related stigma compared to those unemployed. Individuals who perceived their HIV risk as low were less likely to report high HIV-related stigma levels (*p* < 0.001).

**Table 2.** Univariate and multivariate level analysis of factors associated with high stigma level.

Factor	Univariate analysis			Multivariate analysis		
	Crude PR**	95% CI	*p*-value	Adjusted PR	95% CI	*p*-value
Gender						
Female	1			1		
Male	0.91	0.81–1.02	0.113	1.04	0.93–1.15	0.503
Age group						
<25 years	1			1		
≥25 years	0.96	0.85–1.08	0.509	0.97	0.87–1.08	0.567
Marital status						
Single	1			1		
Cohabiting/married	1.02	0.90–1.16	0.712	1.14	1.02–1.28	0.018
Divorced	1.49	1.17–1.90	0.001	1.38	1.07–1.78	0.012
Employment status						
Unemployed	1			1		
Employed	0.81	0.72–0.90	0.001	0.78	0.71–0.87	<0.001
Education level						
Primary	1	–	–	1		
Secondary	1.16	1.00–1.35	0.043	1.01	0.89–1.14	0.891
Tertiary	1.27	1.08–1.48	0.002	1.04	0.91–1.18	0.560
HCT awareness						
No	1			1		
Yes	1.17	1.00–1.36	0.044	0.85	0.75–0.97	0.016
Perceived benefit for HIV testing						
High	1			1		
Low	0.51	0.44–0.60	<0.001	0.69	0.60–0.79	<0.001
Perceived risk for HIV						
High	1			1		
Low	0.32	0.29–0.38	<0.001	0.36	0.31–0.42	<0.001
Health worker effect*						
None	1			1		
Doctors	0.80	0.65–1.00	0.045	0.97	0.77–1.22	0.769
Counsellors	0.95	0.80–1.13	0.565	1.10	0.89–1.34	0.379
Nurses	0.74	0.58–0.96	0.023	1.11	0.88–1.39	0.389

*Health worker responsible for reducing willingness to test for HIV, **PR-prevalence ratio.

Table 2 outlines factors that predicted high HIV-related stigma in the study population. Overall, being married/cohabiting (APR: 1.14 95% CI: 1.02–1.28), divorced (APR: 1.38 95% CI: 1.07–1.78) were associated with high levels of stigma to the disclosure of HIV status. Employed individuals (APR: 0.78, 95% CI: 0.71–0.87), those with a low perception of HIV risk (APR: 0.36 95% CI: 0.31–0.42), are aware of HCT services (APR: 0.85 95% CI: 0.75–0.97) and those who lowly perceived HIV testing as beneficial (APR: 0.69 95% CI: 0.60–0.79) were less likely to report a high level of HIV-related stigma.

**Figure 1.**
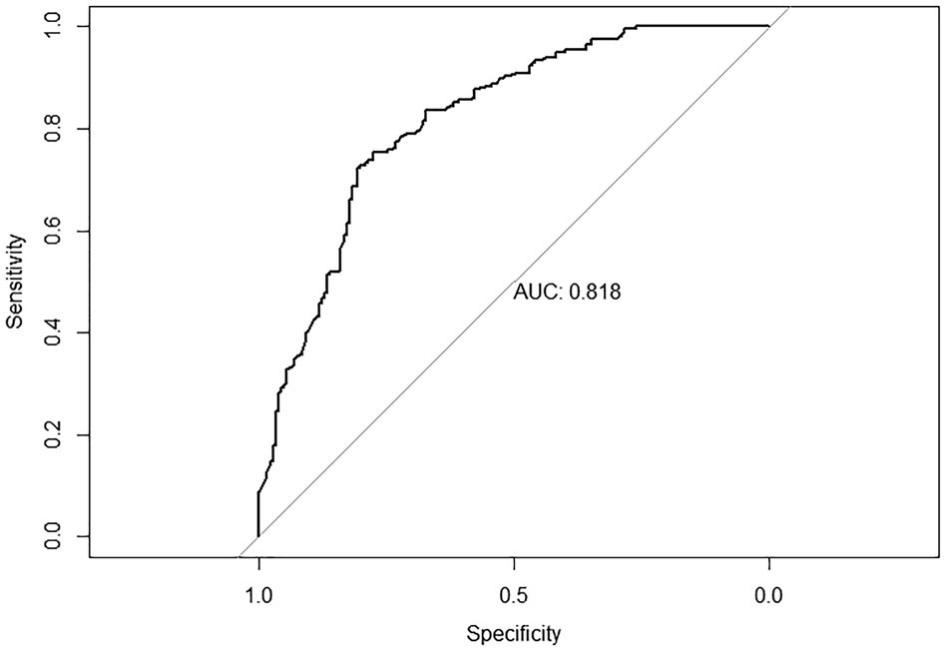
ROC curve.

**Figure 2.**
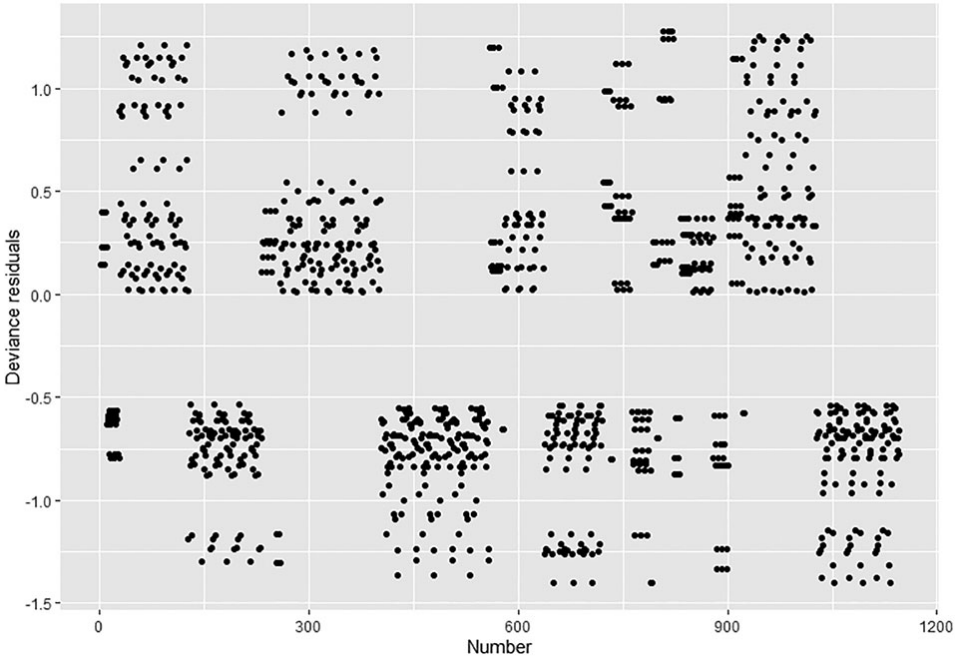
Deviance residuals.

Model diagnostics, ROC curve showed an AUC of 0.81 suggesting a better model with the ability to discriminate high and low stigma (Figure 1). None of the deviance residuals were greater than two (2) suggesting better model fit (Figure 2). Hosmer-Lemeshow test was done and a *p*-value of 1 was obtained suggesting that the model adequately fits the data. Furthermore, no multicollinearity between the independent variables was detected since the tolerance was above 0.10 for all the independent variables. The variance inflation factor also supported that there was no severe multicollinearity since all the values were around 1 (Table 3).

**Table 3.** Assessment of multicollinearity.

Variable	Variance inflation factor	R-squared	Tolerance
Gender	1.12	0.1	0.9
Age group	1.13	0.11	0.89
Knowledge of health centres	1.04	0.03	0.97
Marital status	1.13	0.12	0.88
Education level	1.04	0.03	0.97
Employment status	1.21	0.17	0.82
Health worker stigma	1.02	0.02	0.98
Perceived benefit of HIV test	1.14	0.12	0.88
Perceived HIV infection risk	1.44	0.31	0.69

## Discussion

This study demonstrated a high levels of HIV-related stigma were associated with participants with post-primary education, HIV testing over a year ago and an encounter with a nurse or a doctor at a health facility. In addition, a high level of stigma was associated with being divorced, married and awareness of HIV counselling and testing services. However, the results showed that employment and low perception of HIV risk were protective against high HIV-related stigma.

Low perception about HIV could influence how an individual or community readily embraces an HIV prevention program or access to HIV treatment. Several factors are associated with HIV-related stigma perceptions. A study conducted in Tanzania (Amuri et al., 2011) reported that poverty, less education and living in rural area were associated with high HIV-related stigma. Contrary to these findings, this study did not find an association between low educational level and high HIV-related stigma. This could be due to the fact that this study associated stigma with so many drivers including fear, shame, discrimination, rejection and prejudice against perceived HIV/AIDS-affected individuals, whereas the Tanzania study used only one indicator of HIV/AIDS-related stigma ‘a punishment from God’. The outcome of the study would have been different if other indicators of stigma were evaluated. Interestingly our study finding was also inconsistent with Lim et al., where higher education was associated with low levels of HIV-related stigma (Lim et al., 2013).

Ekstrand et al. reported high HIV-related stigma associated with health care workers including doctors, nurses and ward staff in two cities in India (Ekstrand et al., 2013). The findings were consistent with previous reports showing that an encounter with a healthcare worker such as a doctor or a nurse was associated with a high level of stigma. Stigma reduction programmes in healthcare facilities are needed to improve the quality of healthcare provided to HIV/ AIDS patients.

An essential aspect of HIV prevention is voluntary counselling and testing for people to know their status. However, the stigma associated with HIV could deter people from knowing their status. Individuals with unknown status could potentially have a high HIV-related stigma as previously reported by Kalichman and Simbayi (2003). This also emphasises the need for access to HTS where individuals receive HIV counselling and testing.

Stigma, if not addressed, is likely to negatively affect the 90–90–90 goal of achieving HIV control by 2030 as individuals are prevented from accessing testing services for fear of discrimination and ostracisation. Stigma affects HIV prevention efforts by preventing HIV-positive individuals from developing an adequate support network for fear of rejection by family or friends. Therefore, intervention programs targeting stigma reduction have the potential to positively influence HIV prevention and treatment access in HIV-positive individuals.

### Limitations of the study

The study is a venue-based intercept survey that had some weaknesses. A single-venue taxi rank sampling strategy was considered representative of the City of Johannesburg inhabitants. The use of personal tablets as data collection tools could have prevented potential technophobic participants from answering the survey. This study is from self-reported data and could therefore lead to social desirability bias. However, to mitigate this, the questionnaire had questions that would cross-reference and validate other self-reported questions. In view of these limitations, caution should be exercised in generalising the results to other population groups.

## Conclusions

To effectively control HIV transmission, there is the need to identify strategies to address stigma and other barriers to HIV testing among the commuter population and increase uptake of HCT in this population. This study highlights that a home or mobile outreach centre is the preferred HIV testing place. Therefore, encouraging more HIV testing in communities through mobile outreach centres such as around transport terminus or home-based care need to be considered as possible approaches that could eliminate HIV/AIDS-related stigma.

**Abbreviations**
AIDS: Acquired Immunodeficiency Syndrome
AOR: Adjusted Odds Ratio
CBD: Central Business District
CIs: Confidence Intervals
HIV: Human Immunodeficiency Virus
HTS: HIV Testing Services
HCT: HIV Counselling and Testing
UNAIDS: United Nations Programme on HIV/AIDS
